# Explanatory

**Published:** 1883-06

**Authors:** 


### EXPLANATORY.

In the confusion of the moving of this journal to Buffalo a part of the subscription list was misplaced, and it only turned up some time after most of the edition was mailed. Any of our subscribers, therefore, who received the May number unusually late, will be kind enough to ascribe it to the proper cause. We hope to get everything in proper order after a while, but we must ask that forbearance be exercised for a time, toward both the business and editorial departments.

